# Supplementary material for: A Group-Theoretic Approach to the Origin of Chirality-Induced Spin-Selectivity in Nonmagnetic Molecular Junctions
Source: ACS Nano. 2023 Mar 22;17(7):6452–65. doi: 10.1021/acsnano.2c11410 (PMC10100547; doi:10.1021/acsnano.2c11410)
Supplement: Supplementary file 1 — nn2c11410_si_001.pdf [file nn2c11410_si_001.pdf]

A Group-Theoretic Approach to the Origin of Chirality-Induced Spin Selectivity in Non-Magnetic Molecular Junctions —SI

W. Dednam,[*,†,‡] M. A. García-Blázquez,[¶,‡] Linda A. Zotti,[§,‖] E. B. Lombardi,[†] C. Sabater,[⊥,⊥] S. Pakdel,[#] and J. J. Palacios[@,¶]

† *Department of Physics, Florida Science Campus, University of South Africa, 1710 Johannesburg, South Africa*

‡ *These authors contributed equally to this work*

¶ *Departamento de Física de la Materia Condensada, Universidad Autónoma de Madrid, E-28049 Madrid, Spain*

§ *Departamento de Física Teórica de la Materia Condensada, Universidad Autonoma de Madrid, E-28049 Madrid, Spain*

‖ *Condensed Matter Physics Center (IFIMAC), Universidad Autónoma de Madrid, E-28049 Madrid, Spain*

⊥ *Departamento de Física Aplicada and Unidad asociada CSIC, Universidad de Alicante, E-03690 Alicante, Spain*

CAMD, Department of Physics, Technical University of Denmark, 2800 Kgs. Lyngby, Denmark

@ *Instituto Nicolás Cabrera (INC) and Condensed Matter Physics Center (IFIMAC), Universidad Autónoma de Madrid, E-28049 Madrid, Spain*

E-mail: dednaw@unisa.ac.za

SOC-corrected DFT transport calculations

Pakdel et al.[1] developed a convenient method to correct the band structure of solid materials for spin-orbit coupling (SOC). The core feature of this approach is that quality SOC-corrected bands can be obtained if an existing (or optimized) Gaussian-type orbital (GTO) basis set already reproduces the non-SOC band structure of a material to a good degree, so that SOC can be treated perturbatively. Once that condition is met, adding SOC as a correction in a post self-consistent-field (SCF) step gives good bands in comparison to reference methods which are known to generate very high quality SOC bands by fully-relativistic self-consistent approaches, e.g., `VASP`,[2] `Wien2k`,[3] `Quantum Espresso`[4] or `OpenMX`.[5,6] The latter is used as the reference method in this work.

Here we very briefly outline the method developed by Pakdel et al.[1] in addition to a few recent improvements we have made to it. We use the standard atomic SOC matrix to express the lowest-order Dirac-Kohn-Sham Hamiltonian as follows:

$$\xi\left(r\right)\mathbf{L}\cdot\mathbf{S}=\left[\xi_{\mathrm{ij}}\left\langle l_{\mathrm{i}};m_{l_{\mathrm{i}}};s|\mathbf{L}\cdot\mathbf{S}|l_{\mathrm{j}};m_{l_{\mathrm{j}}};s'\right\rangle\right], \tag{S1}$$

where:

$$\xi_{\mathrm{ij}}=\frac{e^2}{2m_e c^2}\int_0^\infty \frac{1}{r}\frac{\mathrm{d}V_{\mathrm{eff}}\left(r\right)}{\mathrm{d}r}R_{\mathrm{i}}\left(r\right)R_{\mathrm{j}}^*\left(r\right)r^2\mathrm{d}r. \tag{S2}$$

The above simplification relies upon the orthogonality of the radial and angular parts of wave functions in atomic-orbital based DFT, e.g., the Gaussian-type orbitals (GTOs) used by `CRYSTAL14`[7] or `GAUSSIAN09`.[8]

The effective nuclear potential in Eq. (S2) is defined as $V_{\mathrm{eff}}\left(r\right)=-\frac{Z}{r}$,[1] with Z the atomic number. The radial wave functions $R_{\mathrm{i}}\left(r\right)$ are (un)contracted GTOs (CGTOs). The fact that SOC is an intra-atomic phenomenon means that only CGTOs on the same atom and of the same shell type ($L=1$, 2 or 3, referring to P, D, or F shells, respectively) contribute to the integral in Eq. (S2).[1] Note that, due to the lack of adequate nodal structure near the nucleus in pseudopotentials, CGTO basis sets with pseudopotentials require a multiplicative

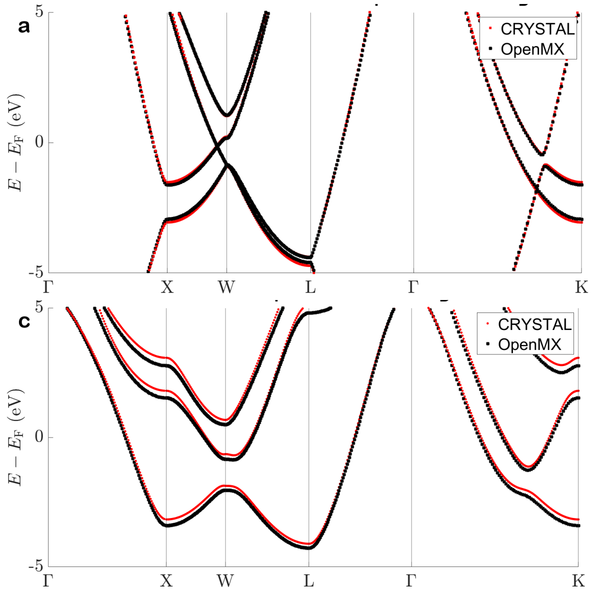

Figure 1: SOC-corrected bands obtained from previous non-SOC calculations in `CRYSTAL14` using our method (solid red markers) and the reference method `OpenMX` (black crosses) for FCC **a** Al, **b** Au, **c** Pb and **d** BCC W.

correction to ξ_{ij} if $V_{\text{eff}}(r)$ is to reproduce the correct effective charge of a given atom.

We describe two minor modifications that improve upon the method described in Ref.[1] First, we have replaced the original effective nuclear potential by a Yukawa screening potential:

$$V_{\text{eff}}(r) = \begin{cases} \frac{-(Z-1)\left[\exp\left(-\frac{lnZ}{r_c}r\right)+1\right]}{r} & r \leq r_c \\ \frac{-1}{r} & r > r_c \end{cases} \tag{S3}$$

where r_c is a cutoff, typically the size of an atomic radius ($\sim 2.5 - 3.0$ a.u.).

Secondly, we do not use a single global multiplicative factor to account for the lack of nodal structure when basis sets with pseudopotentials are used. Instead, we have implemented a multiplicative factor for each shell type (P, D, or F) since it is known that the radial SOC coefficients of different angular momentum shells are multiples of each other.[9]

The ultimate goal of fitting SOC corrected bands to a reference method is to choose high quality basis sets that can be used in DFT quantum transport calculations where transition metal elements, sometimes with strong SOC, are used as the electrodes. We therefore need

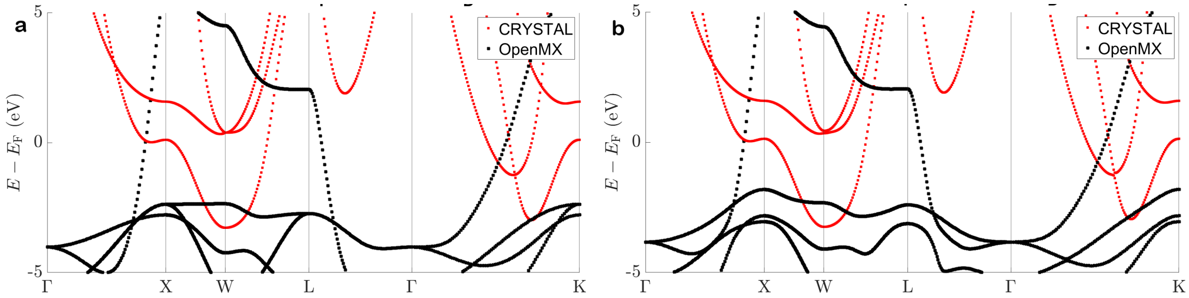

Figure 2: **a** Non-SOC and **b** SOC-corrected bands obtained in `CRYSTAL14` calculations using the unoptimized `x2c-SVPall-2c` all-electron basis set for FCC Au in our method (solid red markers) and a large pseudo-potential basis set in the reference method `OpenMX` (black crosses). The fit is very poor and the addition of SOC does not seem to affect the electronic structure about the Fermi level in our method while the reference method clearly exhibits the lifting of degeneracies expected for a strong SOC metal such as Au and reproduced by our method too in Figure 1. The reference bands in **b** differ slightly from those of Figure 1b because a larger lattice parameter was required to converge the calculation using the all-electron basis set.

such GTO basis sets for metals like Al, Au, Pb, and W, which are used as electrode and/or substrate materials in scanning tunneling microscopy (STM) or mechanically controllable break junction (MCBJ) experiments.[10] In this work, the basis sets used in our electronic transport calculations in the main manuscript have also been employed in band structure calculations in `CRYSTAL14` to make Figure 1, which shows an empirical fit of SOC-corrected bands to `OpenMX` band structure calculations for Al, Au, Pb and W. In all electronic structure and transport calculations we use the `PBE` exchange-correlation functional[11] and strict convergence criteria of at most 10^{-7} Ha. For an all-electron basis set, the multiplicative factors (P, D, or F) per shell should all be set equal to a value of 1 as in the calculation for FCC Al shown in Figure 1a where we used a high quality all-electron basis set which has been optimized for the metallic phase of Al.[12] For Au and W we used basis sets from Ref.[13] with P, D, and F SOC factors of 260, 40, and 10 for Au, and 210, 30, and 10 for W, whilst for Pb we used the basis set from[14] with P, D, and F SOC factors of 280, 30, and 10. All basis sets required optimization of their outer diffuse GTOs in order to get the best empirical agreement with the reference method. We used the `billy` optimizer developed by Mike Towler for this purpose.[15]

The implementation of SOC in our DFT electronic transport method is discussed in detail in other works.[16,17] We merely mention here that the code Atomistic NanoTransport (`ANT.Gaussian`),[18,19] interfacing with `Gaussian09`,[8] has been employed in this work. Out of the box, it allows performing scalar-relativistic, spin-unrestricted calculations. The SOC correction as implemented in `ANT.Gaussian` is freely available online.[20]

It should be mentioned that the small polarizations obtained across a wide energy range about the Fermi level in Ref.[21] in their Au–molecule–Au calculations using an all-electron basis set such as `x2c-SVPall-2c`, if not optimized for the solid phase of the pure metal, may be the result of SOC not having any real effect on the electronic structure of the metal nanofragments that are used as electrodes. The bands without and with SOC in Figure 2a, b respectively, which were obtained from `CRYSTAL14` calculations using the unoptimized all-electron basis set `x2c-SVPall-2c`.

Transversal polarization and $n-$fold rotation symmetry

Here we prove that the spin-polarization is vanishing along any transversal direction if there exists any rotation symmetry $C_{n,l} \in \mathcal{G}$ with $n \geq 2$.

Fixing $\hat{\boldsymbol{z}}$ as an arbitrary transversal direction, we may in principle assign any direction in the $xy-$plane to the longitudinal one, hence we write $\hat{\boldsymbol{e}} = (\sin(\varphi), \cos(\varphi), 0)$, for any $\varphi \in [0, 2\pi)$. Then by equation (4) of the main text:

$$\mathcal{D}^{1/2}(C_n^m) = \begin{pmatrix} \cos(\pi m/n) & \sin(\pi m/n)e^{i\varphi} \\ -\sin(\pi m/n)e^{-i\varphi} & \cos(\pi m/n) \end{pmatrix} \quad , \quad \forall m = 1, \ldots, n-1$$

and by equation (5);

$$G_{\uparrow\uparrow} = \frac{e^2}{h}\sum_{i=1}^{M_A}\sum_{j=1}^{M_B}\left|\cos(\pi m/n)^2 S_{\uparrow\uparrow}^{\beta_j\alpha_i} + \sin(\pi m/n)^2 S_{\downarrow\downarrow}^{\beta_j\alpha_i} + \cos(\pi m/n)\sin(\pi m/n)\left[e^{i\varphi}S_{\downarrow\uparrow}^{\beta_j\alpha_i} + e^{-i\varphi}S_{\uparrow\downarrow}^{\beta_j\alpha_i}\right]\right|^2 ,$$

$$G_{\uparrow\downarrow} = \frac{e^2}{h}\sum_{i=1}^{M_A}\sum_{j=1}^{M_B}\left|\cos(\pi m/n)\sin(\pi m/n)e^{i\varphi}\left[-S_{\uparrow\uparrow}^{\beta_j\alpha_i} + S_{\downarrow\downarrow}^{\beta_j\alpha_i}\right] - \sin(\pi m/n)^2 e^{i2\varphi}S_{\downarrow\uparrow}^{\beta_j\alpha_i} + \cos(\pi m/n)^2 S_{\uparrow\downarrow}^{\beta_j\alpha_i}\right|^2 ,$$

$$G_{\downarrow\uparrow} = \frac{e^2}{h}\sum_{i=1}^{M_A}\sum_{j=1}^{M_B}\left|\cos(\pi m/n)\sin(\pi m/n)e^{-i\varphi}\left[-S_{\uparrow\uparrow}^{\beta_j\alpha_i} + S_{\downarrow\downarrow}^{\beta_j\alpha_i}\right] + \cos(\pi m/n)^2 S_{\downarrow\uparrow}^{\beta_j\alpha_i} - \sin(\pi m/n)^2 e^{-i2\varphi}S_{\uparrow\downarrow}^{\beta_j\alpha_i})\right|^2 ,$$

$$G_{\downarrow\downarrow} = \frac{e^2}{h}\sum_{i=1}^{M_A}\sum_{j=1}^{M_B}\left|\sin(\pi m/n)^2 S_{\uparrow\uparrow}^{\beta_j\alpha_i} + \cos(\pi m/n)^2 S_{\downarrow\downarrow}^{\beta_j\alpha_i} - \cos(\pi m/n)\sin(\pi m/n)\left[e^{i\varphi}S_{\downarrow\uparrow}^{\beta_j\alpha_i} + e^{-i\varphi}S_{\uparrow\downarrow}^{\beta_j\alpha_i}\right]\right|^2$$

Inserting these in equation (1) we obtain, after some algebra:

$$\begin{aligned} P_z = \cot(\pi m/n)\frac{e^2}{h}\sum_{i=1}^{M_A}\sum_{j=1}^{M_B}\left[e^{i\varphi}\left(S_{\uparrow\uparrow}^{\beta_j\alpha_i *}S_{\downarrow\uparrow}^{\beta_j\alpha_i} + S_{\uparrow\downarrow}^{\beta_j\alpha_i *}S_{\downarrow\downarrow}^{\beta_j\alpha_i}\right) + \text{c.c.}\right] = \\ \cot(\pi m/n)\,\mathrm{Re}\left[e^{i\varphi}(P_x + iP_y)\right] \quad , \quad \forall m = 1,\dots,n-1 \end{aligned} \tag{S4}$$

where we have introduced the perpendicular components of the spin-polarization ($\hat{\boldsymbol{z}}$ being the fixed quantization axis, along the chosen transversal direction in this case). Since $\cot(x) = -\cot(\pi - x)$, applying equation (S4) for $m = 1$ and $m = n-1$ (that is, for $C_{n,l}$ and its inverse $C_{n,l}^{n-1}$) we complete the proof.

This result was straightforward to obtain for $n = 2$ since $\mathcal{D}^{1/2}(C_{2,l})$ has two null entries. In that case $C_{2,l}$ is its own inverse and $\cot(\pi/2) = 0$ readily. For $n \geq 3$, however, it must be $\mathrm{Re}[e^{i\varphi}(P_x + iP_y)] = 0$; which is consistent with the fact that in this case the polarization is a vector along the direction of transport, noting that $\varphi = 0$ $(\pi/2)$ if $\hat{\boldsymbol{y}}$ ($\hat{\boldsymbol{x}}$, respectively) corresponds to the longitudinal direction.

On the carbon helix molecules

In the calculations on chiral molecules in Figure 4 of the main manuscript, we have used the molecule in the supplementary material of Zöllner et al.[21] and modified it in two ways. Technically, the original molecule lacks any unitary symmetry, but, in a way, it is not far

from having a 2−fold transversal rotation symmetry $C_{2,t}$ (perpendicular to the axis of the helix). In Figure 4a-f, the hydrogen atoms and two carbon atoms from one end have been removed in order to make the molecule "more asymmetric". In contrast, the molecule exhibits the $C_{2,t}$ symmetry in Figure 4g-i due to the removal of the hydrogen atoms from both ends. All inset structures in Figures 3 and 4 of the main text have been created with the Open Visualization Tool `Ovito`.[22]

Comment on previous results for achiral molecules

Here we briefly comment the results of Guo *et al.*[23] in the context of the present text's formalism. In that reference, a finite longitudinal spin-polarization was obtained by employing a tight-binding model (where symmetries are implicitly present in the relationships between the numerical parameters) for an achiral hexagonal nanotube between chain electrodes, and displacing a single electrode so that longitudinal symmetry planes are broken. Note the results of Figures 2 and 4 in reference[23] are perfectly consistent with our symmetry results for junctions with achiral molecules. In particular, for their $j = 1, 4$ connections, $\mathcal{G} = C_{2v}, C_{2h}$ respectively, albeit with the rotation axis directed transversally, (so that one symmetry plane is longitudinal in both cases), they find $P_l = 0$. Otherwise, mirror planes determine the reversal of the longitudinal polarization when changing the connection in that system according to longitudinal planes, which was also noted by the authors.

Numerical calculations not included in the main text

Here we present the full polarization results, over a range of energies centred on the Fermi level, and corresponding to Figure 2 of the main text. Figure 3 shows the zero-bias spin-polarization along the direction of transport as a function of energy at various angles of relative rotation for **a** Al(001), **b** Al(111), **c** Au(001), **d** Au(111), **e** W(001), **f** W(-110), **g** Pb(001) and **h** Pb(111) nanocontacts.

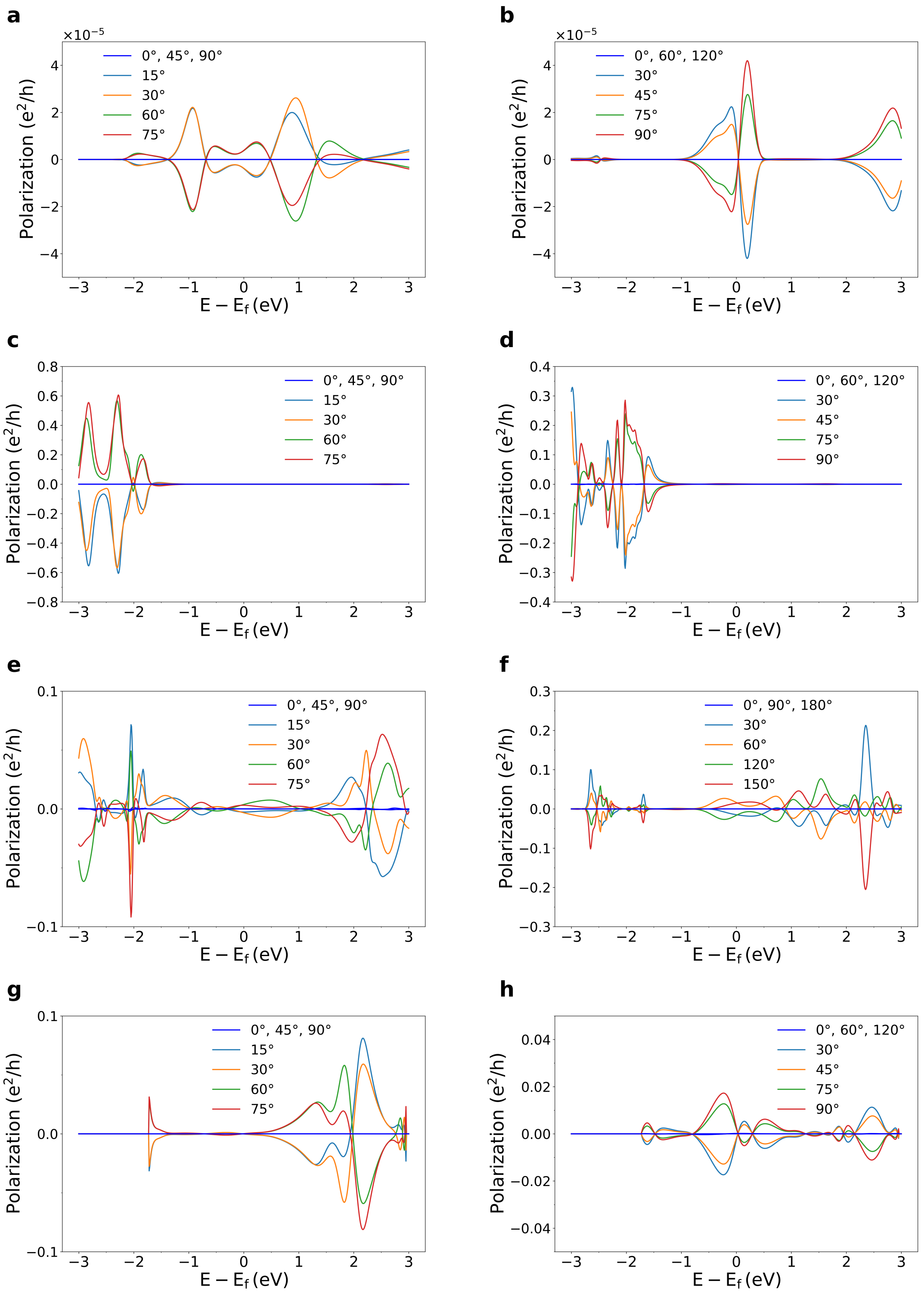

Figure 3: DFT-computed zero-bias spin-polarization along the direction of transport as a function of energy, for several angles of relative rotation between the electrodes (only the top half is rotated). **a**, Al(001). **b**, Al(111). **c**, Au(001). **d**, Au(111). **e**, W(001). **f**, W(-110). **g**, Pb(001). **h**, Pb(111) stacked nanocontacts. Summarized in Figure 2 of the main text.

On the other hand, in Figure 4 we explicitly show that when both longitudinal mirror and rotation symmetries are present in a structure the polarization is vanishing even when the quantization axis is neither parallel nor perpendicular to the direction of propagation (see blue line and inset in blue box in Figure 4), which corresponds to a neither longitudinal nor transversal spin-polarization component. However, when the drain electrode is rotated 15° relative to the source electrode as in the red and green inset boxes, the longitudinal mirror is absent and only rotational symmetry remains, which as we saw in Figure 2a of the main text does not force the polarization to vanish if the spin quantization axis has a projection along the current. The magnitude of the polarization (red) in the structure tilted at 45° with respect to the vertical quantization axis along $\hat{\boldsymbol{z}}$ is reduced relative to the structure oriented along $\hat{\boldsymbol{z}}$.

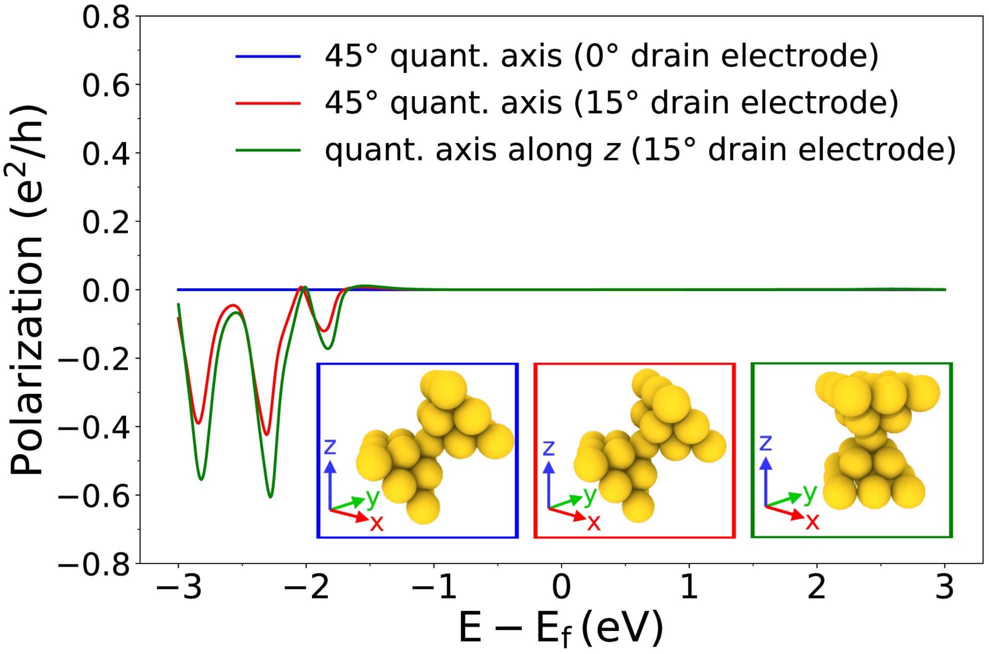

Figure 4: DFT-computed zero-bias spin-polarization along $\hat{\boldsymbol{z}}$ as a function of energy, for Au(001) nanocontacts. The 15° angles in the red and green inset boxes are obtained by rotating the drain electrode with respect to the source electrode.

Finally, in Figure 5 we quantitatively exemplify the impact of defects, in particular atomic

vacancies, on the spin-polarization. The original structure with no defects is a system of bare W(110) nanocontacts with point group $\mathcal{G} = D_{2h}$, so that $\boldsymbol{P} = 0$ as stated in the main text. Upon introduction of random vacancies (N indicating their number), all symmetries were removed at once, enabling a finite $\boldsymbol{P}$ whose component along the direction of transport we plot in the figure. The number of defects is sufficiently low in all cases, taking into account that the original scattering region is formed by 109 atoms, so that the symmetry is preserved at a course scale; but the selection rules for $\boldsymbol{P}$ are indeed determined by the symmetry at the local scale. It should be noted that not all defects will necessarily break all symmetries, or even any symmetry at all. Of course, the nature and position of the defects within a specific system may be important for numerical reasons.

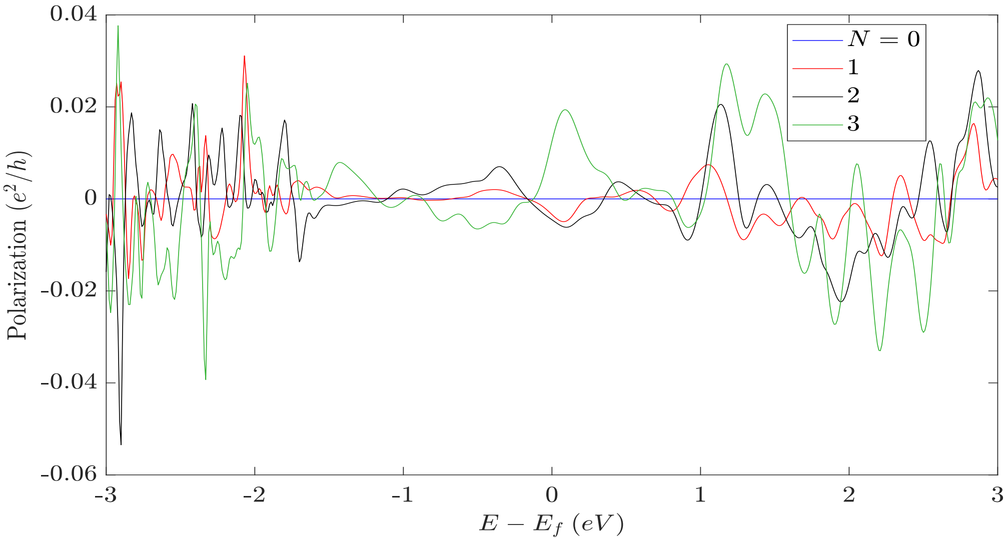

Figure 5: DFT-computed zero-bias spin-polarization along the longitudinal direction as a function of energy, for W(110) nanocontacts (see first column of Figure 1c in the main text) with four atomic layers per contact in the scattering region. N labels the number of atomic vacancies in the structure, which were randomly chosen within the source contact. The corresponding groups are $\mathcal{G} = D_{2h}$ for $N = 0$, and $\mathcal{G} = C_1$ (trivial group) for the other cases.

References

1. Pakdel, S.; Pourfath, M.; Palacios, J. J. An Implementation of Spin–Orbit Coupling for Band Structure Calculations with Gaussian Basis Sets: Two-dimensional Topological Crystals of Sb and Bi. *Beilstein J. Nanotechnol.* **2018**, *9*, 1015.

2. Kresse, G.; Furthmüller, J. Efficiency of Ab-Initio Total Energy Calculations for Metals and Semiconductors Using a Plane-Wave Basis Set. *Comput. Mater. Sci.* **1996**, *6*, 15–50.

3. Blaha, P.; Schwarz, K.; Madsen, G.; Kvasnicka, D.; Luitz, J. *Wien2k: An Augmented Plane Wave+ Local Orbitals Program for Calculating Crystal Properties*; Karlheinz Schwarz, Techn. Universität Wien, Austria, 2001.

4. Giannozzi, P., *et al.* QUANTUM ESPRESSO: a Modular and Open-Source Software Project for Quantum Simulations of Materials. *J. Phys.: Condens. Matter* **2009**, *21*, 395502.

5. Ozaki, T.; Kino, H. Efficient Projector Expansion for the Ab Initio LCAO Method. *Phys. Rev. B* **2005**, *72*, 045121.

6. Ozaki, T., *et al.* *OpenMX (Open Source Package for Material EXplorer)*, Version 3.8. `http://www.openmx-square.org/`: online, 2017; (accessed January 19, 2023).

7. Dovesi, R., *et al.* CRYSTAL14: A Program for the Ab Initio Investigation of Crystalline Solids. *Int. J. Quantum Chem.* **2014**, *114*, 1287.

8. Frisch, M. J., *et al.* *GAUSSIAN09*, Revision C.01, Gaussian, Inc. Wallingford, CT, 2009.

9. Barreteau, C.; Spanjaard, D.; Desjonquères, M.-C. An Efficient Magnetic Tight-Binding Method for Transition Metals and Alloys. *C. R. Phys.* **2016**, *17*, 406.

10. Agraït, N.; Yeyati, A. L.; Van Ruitenbeek, J. M. Quantum Properties of Atomic-Sized Conductors. *Phys. Rep.* **2003**, *377*, 81.

11. Perdew, J. P.; Burke, K.; Ernzerhof, M. Generalized Gradient Approximation Made Simple. *Phys. Rev. Lett.* **1996**, *77*, 3865–3868.

12. Ruiz, E.; Llunell, M.; Alemany, P. Calculation of Exchange Coupling Constants in Solid State Transition Metal Compounds Using Localized Atomic Orbital Basis Sets. *J. Solid State Chem.* **2003**, *176*, 400–411.

13. Laun, J.; Bredow, T. BSSE-Corrected Consistent Gaussian Basis Sets of Triple-Zeta Valence with Polarization Quality of the Sixth Period for Solid-State Calculations. *J. Comput. Chem.* **2021**, *42*, 1064–1072.

14. Zagorac, D.; Doll, K.; Schön, J. C.; Jansen, M. Ab Initio Structure Prediction for Lead Sulfide at Standard and Elevated Pressures. *Phys. Rev. B* **2011**, *84*, 1–13.

15. Towler, M. *billy*, Version 5.0. `https://vallico.net/mike_towler/crystal.html`: online, 2014; (accessed January 19, 2023).

16. Dednam, W. Atomistic Simulations of Competing Influences on Electron Transport Across Metal Nanocontacts. Ph.D. thesis, University of Alicante/University of South Africa, 2019.

17. Dednam, W.; Zotti, L. A.; Pakdel, S.; Lombardi, E.; Palacios, J. Spin-Imbalances in Non-Magnetic Nano-Systems: Using Non-Equilibrium Green's Function DFT to Model Spin-Selective Phenomena Mediated by Spin-Orbit Coupling. *Proc. 65th Annual Conf. South African Institute of Physics* **2022**, *65*, 25–30.

18. Palacios, J. J.; Pérez-Jiménez, A. J.; Louis, E.; Vergés, J. A. Fullerene-Based Molecular Nanobridges: A First-Principles Study. *Phys. Rev. B* **2001**, *64*, 115411.

19. Palacios, J. J.; Pérez-Jiménez, A. J.; Louis, E.; SanFabián, E.; Vergés, J. A. First-Principles Approach to Electrical Transport in Atomic-Scale Nanostructures. *Phys. Rev. B* **2002**, *66*, 035322.

20. Palacios, J. J., *et al.* ANT.Gaussian Release 2.7.1. **2022**, GitHub. `https://github.com/juanjosepalacios/ANT.Gaussian` (accessed January 19, 2023).

21. Zöllner, M. S.; Saghatchi, A.; Mujica, V.; Herrmann, C. Influence of Electronic Structure Modeling and Junction Structure on First-Principles Chiral Induced Spin Selectivity. *J. Chem. Theory Comput.* **2020**, *16*, 7357–7371.

22. Stukowski, A. Visualization and Analysis of Atomistic Simulation Data with OVITO-the Open Visualization Tool. *Modelling and Simulation in Materials Science and Engineering* **2010**, *18*, 015012.

23. Guo, A. M.; Pan, T. R.; Fang, T. F.; Xie, X. C.; Sun, Q. F. Spin Selectivity Effect in Achiral Molecular Systems. *Phys. Rev. B* **2016**, *94*, 1–5.
